# Advances towards the use of gastrointestinal tumor patient-derived organoids as a therapeutic decision-making tool

**DOI:** 10.1186/s40659-023-00476-9

**Published:** 2023-12-02

**Authors:** Javiera Obreque, Luis Vergara-Gómez, Nicolás Venegas, Helga Weber, Gareth I. Owen, Pablo Pérez-Moreno, Pamela Leal, Juan Carlos Roa, Carolina Bizama

**Affiliations:** 1 Department of Pathology, School of Medicine, Pontificia Universidad Católica de Chile, Diagonal Paraguay 362, Office 526, 8330024 Santiago, Chile; 2 Millennium Institute on Immunology and Immunotherapy, Pontificia Universidad Católica de Chile, 8331150 Santiago, Chile; 3 Centre of Excellence in Translational Medicine (CEMT) and Scientific and Technological Bioresource Nucleus (BIOREN), Biomedicine and Translational Research Lab, Universidad de La Frontera, 4810296 Temuco, Chile; 4 Department of Physiology, Faculty of Biological Sciences, Pontificia Universidad Católica de Chile, 8331150 Santiago, Chile; 5 Advanced Center for Chronic Diseases, Pontificia Universidad Católica de Chile, Santiago, Chile; 6 Centro de Prevención y Control de Cáncer (CECAN), Pontificia Universidad Católica de Chile, Santiago, Chile

**Keywords:** Patient-derived organoid, Gastrointestinal cancer, Pre-clinical model, Drug response prediction, Immunotherapy, Tumor microenvironment

## Abstract

In December 2022 the US Food and Drug Administration (FDA) removed the requirement that drugs in development must undergo animal testing before clinical evaluation, a declaration that now demands the establishment and verification of ex vivo preclinical models that closely represent tumor complexity and that can predict therapeutic response. Fortunately, the emergence of patient-derived organoid (PDOs) culture has enabled the ex vivo mimicking of the pathophysiology of human tumors with the reassembly of tissue-specific features. These features include histopathological variability, molecular expression profiles, genetic and cellular heterogeneity of parental tissue, and furthermore growing evidence suggests the ability to predict patient therapeutic response. Concentrating on the highly lethal and heterogeneous gastrointestinal (GI) tumors, herein we present the state-of-the-art and the current methodology of PDOs. We highlight the potential additions, improvements and testing required to allow the ex vivo of study the tumor microenvironment, as well as offering commentary on the predictive value of clinical response to treatments such as chemotherapy and immunotherapy.

## Background

The US Food and Drug Administration (FDA) recently declared that drugs under development may not necessarily require animal testing before human clinical evaluation and subsequent administrative approval [1, 2]. This change, long expected by animal welfare groups, down plays the importance of animal models after more than 80 years of use in drug safety regulation, and has brought to light the need to establish in vitro and ex vivo models to allow drug efficacy and safety testing. Gastrointestinal (GI) tumors, which due to their primarily late stage diagnosis deliver high lethality rates, are central to the search for new therapeutic options. The principal types of GI cancers include colorectal (CRC), gastric (GC), hepatocellular carcinoma (HCC), esophagus (EAC), pancreas (PDAC) and gallbladder (GBC). According to GLOBOCAN 2020 global statistics, there are an estimated 5.1 million new GI cancer cases per year (26,7% of the global incidence) and 3.6 million (36.4%) deaths [3]. Although the incidence of some GI cancer has decreased in recent decades, this group of malignancies continues to be a major burden to public health [4]. In advanced stage, combined surgery with the administration of chemotherapy and/or radiotherapy still only offers an 5-year overall survival rate of less than 15%, principally due to the high resistance and the tumor aggressiveness [5]. Encouragingly, biological therapies in the form of HER2 and VEGFR-2 inhibitory antibodies are now available to subgroups of GI malignancies. Furthermore, immune checkpoint blockage has recently started to show improvement in quality of life and overall survival, both in refractory malignancies and first line treatment [5, 6]. However, due to differential response brought about by the tumor heterogeneity, new therapeutic strategies for GI cancers patients are required and a new preclinical platform that recapitulates and anticipates therapeutic response is urgently needed.

For this purpose, patient-derived organoids (PDOs) have emerged as robust preclinical models in precision medicine and provide several advantages over pre-existing models. In their ability to recapitulate the molecular and cellular characteristic of the original tumor, the PDOs have been used as a platform for both drug screening and predicting patient response to chemotherapy, targeted therapy and immunotherapy. It would be desirable to create biobanks of cancer samples that give rise to PDOs to allow the delivery of reproducible models for drug screening in the delineation of novel therapeutic strategies. Herein, we review the current models and features of GI-Tract PDOs and highlight the potential of this ex vivo platform in drug screening and clinical prediction. Towards a future clinically viable alternative to animal testing, we will outline the advances, challenges and requirements in incorporating the multifaceted aspects of the tumor microenvironment (TME) in a GI tract PDO model.

## Preclinical models using in GI tract cancer research

As in the majority of neoplasms, the variable sensitivity to anticancer treatments have been observed among GI cancer patients, which reflects the high cellular and molecular heterogeneity present in the tumor bulk [7]. The majority of models used in preclinical cancer research to date have shown low reproducibility when implemented in clinical settings [8]. The failure of traditional study models to mimic native tumor biology is no doubt multifactorial. Deficiencies may lie in the diversity of signaling pathways altered in tumorigenesis, the failure to fully recapitulate the TME, the presence of the deaminating function of the liver and the activation of the inflammatory and immune systems [8, 9]. Thus, new platforms that maintain pathological and altered driver pathways similar to the in vivo tumor are urgently required if clinical response is to be predicted ex vivo.

Among the many different approaches to predict drug sensibility in human cancers, the approaches of primary cultured fresh surgical tissue, the use of immortalized cancer cell lines grown on plastic (2D) and the xeno-transplantation of tumors into immunodeficient mice (PDXs) have been among the most widely used. Primary culture of tumor cells or tissue is an in vivo or ex vivo model capable of maintaining a relatively stable epithelial phenotype [10]. Aziz and colleagues established a method for in vitro primary cultures from fresh gastric surgical tissues that was able to recapitulate the purity of a mucin secreting gastric epithelial phenotype [10]. However, the main disadvantage of primary culture is the short-term and low passage number in which tumor heterogeneity is maintained and the inevitable establishment of subclones. Moreover, the rapid overgrowth of fibroblasts hinders the preservation of cancer cells and thus alters the translational value of these models [10, 11]. Every model has its advantage and for immortalized cancer cells lines grown in 2D in vitro there is the option of long-term culture at a relatively low cost, easy experimental manipulation and good reproducibly in growth and response [12]. At low passage number gastrointestinal cancer cell lines have been able to retain the genotype and phenotype of their originating tumor sample which makes then useful for exploratory science and initial high-throughput screening approaches, however, as every cancer is unique, this model does not offer individual patient treatment prediction [12]. The long-term serial passaging of cell lines have been associated which the loss of cancer-specific heterogeneity through the gradual epigenetic and genetic drift and the upregulation of genes that facilitate the survival and multidrug resistance [13–16]. A further drawback of in vitro 2D approaches comes changes in mechanobiology brought about by attachment to a rigid dish (typically plastic or glass) that discourages cell-to-cell interactions and hinders the complex layering and structural formation that is present within the 3D organization of tissues and tumors alike within the body. In contrast, PDXs generated by directly transplanting a fragment of patient tissue into immunodeficient mice can recapitulate better the histology and the inter- and intra-tumoral genetic and functional heterogeneity of the in vivo gastrointestinal TME [17]. Moreover, PDXs have emerged as a valuable preclinical tool for studying tumor progression and metastasis and as models to predict response to oncology treatments [18, 19]. This PDX model reflects the cellular interaction between malignant cells and TME, recapitulating histological, genetic and functional heterogeneity of the in vivo gastrointestinal tumor. While representing a faithful preclinical model to study metastasis-related features [17, 19], the PDX can also suffer clonal selection and diverge from the original tumor characteristics and thus limit translational applications to only early-passage numbers [20, 21]. The xenograft model of human cancer cells requires the use of immunosuppressed mice. This removes the essential involvement of the immune system in tumor growth and drug response, and obviously removes any possibility of immunotherapy screening. An alternative is to use an orthotopic model (animal tumors in the animal of origin); however, this does not aid in defining a personalized therapy for a cancer patient. Furthermore, the PDX model is labor and resource-intensive due to the requirement of extensive mice colonies (which in turn may present bioethical dilemmas). Furthermore, the 4–8 months required to gain sufficient material, and the non-existent possibility to implement high throughput screening, may discourage the development of this model as a clinical decision-making tool [22, 23], especially when the FDA is currently promoting the reduction in animal use [2]. However, the PDXs are feasible and cost-effective models for final in vivo validation platforms, providing valuable information about promissory treatments.

A third cancer model reported to have clinical utility is the three-dimensional (3D) culture system of human tumors, of which spheroids and patient-derived organoids (PDOs) are the most reported to date. Although 3D cultures are rapidly expanding to bridge the gap between cell culture and animal models, the all-encompassing use of the term “3D culture” often does not bring to the fore the important differences in complexity and distinct biological purpose between the culture systems. Spheroids are a simple clusters of enriched free-floating stem-like cancer cells, whose low complexity limits their use in mirroring tumor organization, even when grown in hydrogel homologous to the ECM [24]. The majority of the GI cancer spheroids are derived from either 2D commercial cell lines (cell line-derived organoids) or xenographs [25–27]. However, there are reports in which CRC and GC spheroids have been established from primary tissues and these cultures frequently retain the tumor heterogeneity [25, 28]. These cultures, that can be maintained for a short time in culture, only partially mirror tumor organization and do not fully reflect patient-specific organotypic clusters [24, 29, 30]. In contrast, PDOs are 3D tissue cultures of longer duration that resemble the epithelial cell conglomerates derived from the proliferation and differentiation of adult cancer stem cells (CSC) present in a specific organ [29]. To obtain 3D organization, stem cells need to be stimulated and grown in the presence of a critical component cocktail of R-Spondin-1 (Rspo-1) to facilitate intestinal epithelial growing from Lgr5^+^ crypt base columnar cells [31], epidermal growth factor (EGF) to promote proliferation, and Noggin to act as a bone morphogenic protein antagonist in a matrix that mimics the basal lamina. The stem cells are embedded within a natural extracellular matrix (ECM), typically Matrigel or similar commercial matrix, which acts as a scaffold to promote the formation of cell–cell adhesions and 3D expansion [32]. This organotypic system can retain with high fidelity the in vivo tumor epithelium and pathophysiological features such as disease heterogeneity, histological architecture, degree of tumor differentiation, mutational landscape and molecular expression profile [22, 33]. A disadvantage of organoids it is unique epithelial nature, making it difficult to assess the effect of treatment targeting in non-epithelial cells, such as endothelial or immune cells [34]. Regardless of this drawback, the PDO 3D model overcomes the limitations of the traditional cancer models as it can be established in a relatively short time period, it is easy to manipulate and it reproduces the complex spatial morphology facilitating high-throughput screening [35]. The principal characteristics of the most promising GI-tract preclinical models are presented in Table 1**.** Overall, investigation to date suggests that PDOs represent a suitable ex vivo model with preclinical utility, which could enhance the translational application of a plethora of potential therapies and offer correlation with clinical outcomes.

**Table 1:** Characteristics of the preclinical models used in cancer research

Features	Patient-derived tumor cell culture	Patient-derived xenograph (PDX)	Patient-derived spheroids	Patient-derived organoids (PDOs)
Time to model establishment	24–48 h	Variable, 2–6 week [108]	1 day [25]	2–3 week [47]
Diameter	10–30 µum [12]	Until 1–2 mm^3^ (then passages)	20–540 µm [25, 109]	200–500 µm
Establishment efficiency^a^	Low, 10–15% CCR [110]; 42.1% GC ascites fluid [111]	19% GIST [112]; 54.8% GIs [112, 113]; 15.1 – 34.1% GC [111, 114]; 59% PDAC; 86% and 35% GBC [115]	47% [25]; 80% GC [28]	> 50% GIs cancers
Cost	Cheap (culture medium, 1 × penicillin/streptomycin and 5–10% fetal bovine serum)	Expensive (Mouse Facility, ECM, fresh tissue from GIs patients)	Moderate (DMEM/F12 medium supplemented with B27, bFGF, EGF and Insulin in ultralow attachment plates) [109]	Expensive, Commercial mediums or WNR-conditioned medium; 50% ECM; antibacterial and antifungal compound, B27 and N2 Supplements; specific recombinants growth factors and inhibitors; fresh tissue from GIs patients
Representativeness from parental tumor	Variable, more in low-passages (1–4) [12]	Very good, also recapitulated the TME interaction and metastasis process	Variable, retain phenotype and genetic from parent tissues. Promote expansion CSC [25, 28]	Good, retain genetic and phenotypic heterogeneity
Growth viability	Long-term expansion (> 90 passages, 1 year) [114]	Long-term expansion (~160 days) [114]	Short-term (~10 days)[30]	Long-term expansion (~100 passages, 1 year)[41]

*GIST* gastrointestinal stromal tumors, *GIs* gastrointestinals, *GC* gastric cancer, *PDAC* pancreatic ductal adenocarcinoma, *GBC* gallbladder cancer

^a^Percentage based on number of cases established from total tumor patient tissue processed

## Patient-derived organoids mimic the gastrointestinal primary cancers

**Fig. 1:**
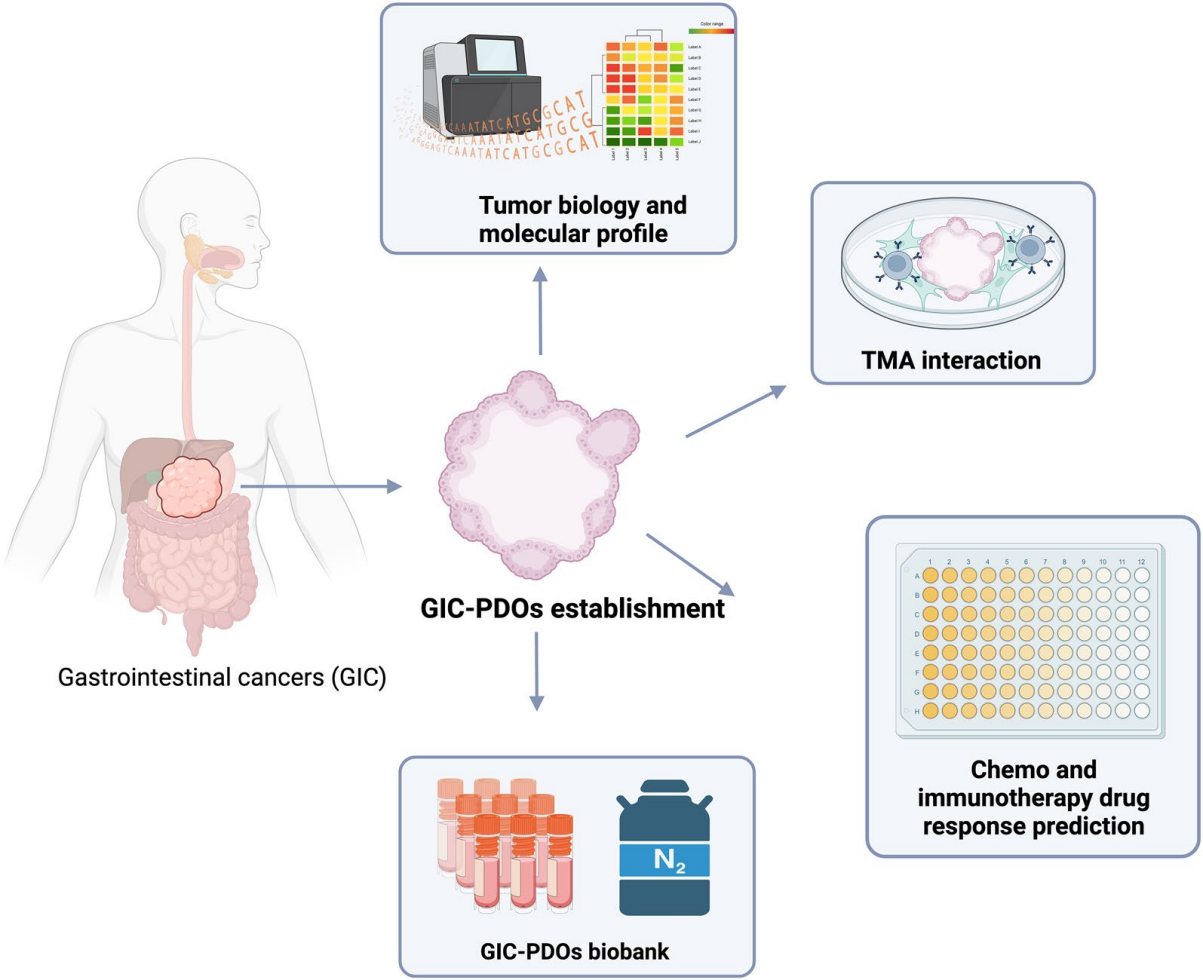
Gastrointestinal cancer PDOs and their main applications. PDOs have a wide range of applications in basic and traslational medicine. Herein, we highlight the PDO platform as a tool for tumor biology and molecular characterization tumor microenvironment (TME) interaction, chemo and inmunotherapy drug response prediction and biobanking. The figure as created with Biorender

GIs PDOs are characterized by retaining the in vivo tumor architecture, the genotypic and phenotypic characteristics and recapitulating the inter-tumor heterogeneity reported in gastrointestinal cancers [36]. Stem cell culture conditions have been successfully adapted to generate PDOs with an establishment efficiency greater than 50% from a variety of normal and tumor GI tissues (Table 2), including colorectal [22, 37–40], liver [41], pancreas [42–44], gastric [45–47], rectal [48] and gallbladder/biliary tract origins [39, 49]. In general, the establishment of gastrointestinal organoids can be obtained with 50–80% of efficiency from fresh primary and metastatic cancer tissues patients. Although the establishment success of PDOs depends on factors such as sample size and percentage of the tumor cells [50], the process requires only a short time in culture (2–4 weeks). Furthermore, the potential is present for multiple passages (at least up to 100) that each maintain the genetic identity of the originating tumor. In a similar fashion to other preclinical models, PDOs also offer the potential to perform the plethora of tumor biology studies. A summary of GI-PDOs applications that we will focus on in this review is shown in Fig. 1. However, of particular interest to the clinic, is the potential that the PDO model can be a useful tool in predicting patient-specific sensitivity to antineoplastic treatments in GI neoplasms [37, 48, 51–53], and accordingly biobanks of PDOs have been established from different GI cancer tissue [37, 40, 54]. As will be discussed in greater detail in the following sections, to address the limitation of an incomplete TME, the co-culture of organoids with other non-epithelial cells has been developed. For example, pancreatic patient derived organoids (PDAC-PDOs) have been co-cultured with cancer-associated fibroblasts (CAFs) to elucidate the role of fibroblasts on drug sensitivity and resistance. Interestingly, co-cultured PDAC-PDOs and CAFs displayed enhanced proliferation, increased drug resistance and a concordant epithelial-mesenchymal transition (EMT)-related gene expression pattern [55]. Similarly, the influence of immune cells has been studied on the malignant properties of other GI PDOs such as colon cancer, gastric cancer and cholangiocarcinoma [56–58]. Additionally, the presence of cancer associated microorganisms on epithelial tissue also has been examined in co-culture [59].

**Table 2:** Studies reporting GI cancers PDOs and their main culture characteristics

GI cancer type	Sample obtention	Method	Main outcomes	References
Colon adenocarcinoma	Surgery resected tissues from 20 patients with colon cancer	*Digestion:* collagenase type IX and dispase type II for 30 min at 37 ℃ *Culture:* 50% Matrigel in basal culture medium (advanced Dulbecco’s modified Eagle medium/ F12 supplemented with penicillin/streptomycin, Glutamax, B27, n-acetylcysteine) supplemented with EGF, gastrin, A83-01, SB202190 without growth factors	Optimization of long-term expansion protocol to colon tumor epithelial PDOs	[60]
Colon adenocarcinoma	22 surgery resected tissues and 19 normal-adjacent were derived from 20 patients with colon tumors	*Digestion:* Collagenase II, hyaluronidase and Ly27632 for 30 min at 37 °C *Culture:* BME with Human Stem cell Medium (HICS) minus Wnt (basal culture medium with 20% Rspo-1 conditioned medium, 10% Noggin conditioned medium, B27, n-acetylcysteine, nicotinamide, hEGF, Gastrin, A83-01, SB202190 and Primocin *Drug screening:* 15–20,000 organoids/ml seeded in 2% BME/growth media and 7-point of 83 compound library drug were tested. At 6 days, the cell viability was analyzed using CellTiter-Glo	CC-PDOs culture was successful in 81,5% (22/27) and closely recapitulate histological and genetic properties of the original tumor PDOs are amenable to high throughput drug screens allowing detection of gene-drug association	[40]
Metastatic colorectal cancers	14 patients’ biopsies from accessible metastatic lesions Clinical trial: NCT01855477	*Digestion:* was performed as described Sato et al. [60] *Culture:* Matrigel and basal culture medium supplemented with 20% Rspo-1 conditioned medium and 10% Noggin conditioned medium, nicotinamide, human EGF, gastrin, A83-01, SB202190, PGE2 and Primocin	CRCPDOs culture was successful in 71% (10 of 14 cases) and 90% of somatic mutations were shared and DNA copy number profiles were correlated between PDOs and biopsies from the same patients	[22]
Colorectal tumors	52 tumor samples, 23 surgery resected tissues and 29 endoscopic biopsies from 43 patients	*Digestion:* was performed as described Sato et al. [60] with several modifications. Liberase digestion for 60 min at 37 °C *Culture:* Matrigel and advanced DMEN/F12 supplemented with penicillin/streptomycin, HEPES, Glutamax, B27, gastrin I, n-acetylcysteine. Niche factors: EGF, Noggin, 10% Rspo-1 conditioned medium and 50% Wnt-3A, A83-01 and SB202190	55 CRCPDOs was successful in 100% of tissues. The PDOs often displayed Wnt or hypoxia dependency or SB sensitivity. The niche factors dependency decreases in adenoma-carcinoma transition	[62]
Metastatic gastrointestinal cancers	Ultrasound, CT-guide and endoscopic biopsies of 16 mCRC; 4 mGOC and 1 mCC from cancer patient. Clinical trials: NCT02994888 and NCT03010722	*Digestion:* PBS/EDTA containing 2 × TrypLe for 1 h at 37 °C *Culture:* Matrigel and organoid medium containing DMEN/F12 medium supplemented with EGF, Noggin, Rspo-1, gastrin I, FGF-10, Wnt-3A, PGE2, Y-27632, nicotinamide, A83-01 and SB202190. HGF only in cholangiocarcinoma organoids *Drug screening:* 4500–6000 cells were seeded in 30% of Matrigel and were treated with 50 custom-made library compounds in technical triplicate at 1 mM concentration. At 6–8 days, the cell viability assayed using CellTiter-Blue	GIs-PDOs culture was successful in 70% of biopsies. 96% overlap in mutational spectrum was observed between PDOs and their parental biopsies 100% sensitivity, 93% specificity, 88% positive predictive value, and 100% negative predictive value in forecasting response to targeted agents or chemotherapy in patients	[52]
Colorectal cancers and paired liver metastasis	72 surgery resected tissues, 36 primary CRC and their liver metastasis matched	*Digestion:* DMEM medium containing collagenase IV, collagenase II, hyaluronidase, dispase type II and YM-27632 for 30 min at 37 °C *Culture:* Medium and Matrigel 1:2 ratio and organoid medium containing DMEN/F12 medium supplemented with Rspo-1, Noggin, EGF, HEPES, Glutamax, Normocin, Gentamicin/amphotericin B, N2, B27, n-acetylcysteine, nicotinamide, A83-01, SB202190, gastrin and PGE2 *Drug screening:* 200 ±50 PDOs cells were seeded in Matrigel and were treated at 7-point concentration of 5-FU, CPT11, oxaliplatin and FOLFOX and FOLFIRI. At 3 days, the cell viability assayed using CellTiter-Glo 3D	PDOs culture was successful in 86,1% of CRC and 75.0% of liver metastasis and capture intra and interpatient heterogeneity at multiomic level PDOs in vitro response correlated with RECISTs and prognosis. PDOs may have a predictive value to determinate the risk of disease progression with FOLFOX and FOLFIRI	[37]
Metastatic colorectal cancers	67 tumor biopsies from 61 patients before start of treatment from TUMOROID clinical trial study (NL49002.031.14)	*Digestion:* Biopsy was dissociated with sharp needles *Culture:* The PDOs were cultured as a previously described in Sato et al. [60] and Dijkstra et al. [103] *Drug screening:* PDOs previously dissociated using dispase II. 100 PDOs per well were resuspended at 1:2 concentration of medium: geltrex. PDOs were incubated in four-fold drug matrices of 5-FU + oxaliplatin or 5-FU + irinotecan and two-fold single-drug doses response curves in technical triplicate. At 72 h of incubation the cell viability was measure using CellTiter-Glo 3D	PDOs culture was successful in 63% DOs predicted response for Irinotecan in more of 80% of patients. The PDOs do not predict response to 5-FU/capecitabine plus oxaliplatin combined therapy	[38]
Rectal cancer	65 rectal cancer PDOs from 58 individual patients collected by surgery and biopsy	*Digestion:* collagenase Type XI and 125 mg/mL dispase type II for 40 min at 37 °C *Culture:* Matrigel and organoid medium containing advanced DMEM/ F12 supplemented with antibiotic–antimycotic, B27, N2, Glutamax, HEPES, N-acetylcysteine, nicotinamide, 50% Wnt3a, 20% Rspo-1 conditioned medium, Noggin, EGF, A83-01, Y-27632 and SB202190 *Drug screening:* 50.000 cells were coated with 50% Matrigel. The PDOs were cultures with different doses to 5-FU, FOLFOX. For radiation experiment, 10.000 cells embedded 30 mL Matrigel and irradiated at 250 kVp and 12 mA. At 6 or 10–13 days, the cell viability assayed using CellTiter-Glo	PDOs overall success rate of 77% (65/84) PDOs retained molecular features of the derived tumors, and their ex vivo responses to clinically relevant chemotherapy and radiation treatment correlated with the clinical responses	[63]
Locally Advanced Rectal Cancer	112 Biopsy tumors of primary rectal cancer tumor, Clinical Trial: NCT02605265	*Digestion:* collagenase IV, collagenase II, hyaluronidase and dispase II (0.1 mg/mL) for 30–60 min at 37 °C *Culture:* Matrigel and organoid medium containing advanced DMEM/ F12 supplemented with Rspo-1, Noggin, EGF, HEPES, Glutamax, Normocin, Gentamicin/amphoteritin B, N2, B27, n-Acetylcysteine, Niacinamide, Alk 4/5/7 inhibitor, p38 inhibitor, Gastrin and PGE2 *Drug screening:* 200 ± 50 PDOs in 15 ml Matrigel with 300 mL medium. PDOs with or without chemotherapy treatment (10 mM 5-FU or 10 mM irinotecan (CPT-11) were exposed to X-rays (246 cGy/min, 250.0 kV, 12.00 mA, SSD = 50 cm). The cell viability was evaluated each three days using CellTiter-Glo 3.0	PDOs culture was successful in 85.7% (96/112) PDOs closely recapitulate the pathophysiology and genetic changes of corresponding tumors Chemoradiation responses in patients are highly matched to RCO responses, with 84.43% accuracy, 78.01% sensitivity, and 91.97% specificity	[48]
Ductal pancreatic cancer	20 primary tumor samples	*Digestion:* collagenase (Roche) *Culture:* Matrigel and tumor organoid medium containing DMEN with B27, ascorbic acid, insulin hydrocortisone, FGF-2, all-trans retinoic acid and Y267632 *Drug screening:* 2,500 cells seeded in Matrigel. At day 1 and 4 inhibitors and gemcitabine were added. At day 8, cell growth was analyzed using CellTiter 96 nonradioactive cell proliferation assay	PDOs culture was successful in 85% (17/20) samples and maintain phenotypic heterogeneity of the primary tumor PDOs retain patient-specific physiologic changes including hypoxia, oxygen consumption, epigenetic marks, and differential sensitivity to EZH2 inhibition	[42]
Liver tumors	Resected HCC (n = 3), CC (n = 3), combined CHC tumors (n = 2) and one healthy-liver derived donor (as a control)	*Digestion:* collagenase D, DNase I for 20–40 at 37 °C *Culture:* BME plus advanced DMEM/F12 supplemented with Penicillin/Streptomycin, Glutamax, 10 mM HEPES, B27 (without Vitamin A), N2, n-acetylcysteine, 10% (vol/vol) Rspo-1 conditioned medium, 30% (vol/vol) Wnt3a conditioned medium, nicotinamide, gastrin I, EGF, FGF-10, HGF, Forskolin, A83-01, Noggin and Y27632 *Drug screening:* 15,000–20,000 organoids seeded with 2% Matrigel. At following day were treated 29 anticancer compound, 4 point for 6 days and cell viability was evaluated using CellTiter-Glo	Establishment of PDOs was 100% and were expanding long-term (~ 1 year) Primary PDOs recapitulate the histological, > 80% of the cancer-related variant and transcriptomic profiles present in the original tumor In vivo xenograft of PDOs showed tumorigenic and metastatic potential PDOs facilitate the prediction of drug sensitivity and/ resistance in patient-specific manner	[41]
Liver tumors	Needle biopsies with ultrasound guidance	*Digestion:* collagenase IV and DNase I at 37 °C *Culture:* BME-2 plus advanced DMEM/F12 supplemented B27, N2, nicotinamide, n-acetylcysteine, gastrin, forskolin, A83-01, EGF, FGF-10, 10% Rspo-1 conditioned medium and 30% (vol/vol) Wnt3a conditioned medium *Drug screening:* 5,000 cells in BME2. At 6-day, sorafenib was added and cell viability was evaluated using CellTiter-Glo	HCC-PDOs with efficiency of generation of 33% (8/24) and retain the morphology and expression of HCC markers and preserve the genetic heterogeneity of the original tumors. HCC-PDOs display variable sensitivity to sorafenib Xenograph implantation in 80% of HCC-PDOs (16/20)	[64]
Ductal pancreatic cancer	20 primary tumor samples	*Digestion:* collagenase (Roche) *Culture:* Matrigel and tumor organoid medium (DMEM with B27, ascorbic acid, insulin hydrocortisone, FGF-2, all-trans retinoic acid and Y267632) *Drug screening*: 2500 cells seeded in Matrigel. At day 1 and 4 inhibitors and gemcitabine were added. At day 8, cell growth was analyzed using CellTiter-Glo 96 nonradioactive cell proliferation assay	PDOs culture was successful in 85% (17/20) samples PDOs maintain phenotypic heterogeneity of the primary tumor and retain patient-specific physiologic changes including hypoxia, oxygen consumption, epigenetic marks, and differential sensitivity to EZH2 inhibition	[42]
Ductal pancreatic cancer	10 human samples from surgical resection	*Digestion:* Collagenase II in human complete medium at 37 ºC for 16 h and treatment with TrypLe for 15 min at 37 ºC *Culture:* Matrigel and DMEM/F12 medium supplemented with HEPES, Glutamax, penicillin/streptomycin, B27, Primocin, n-acetylcysteine, Wnt3a-conditioned medium (50% v/v), Rspo-1conditioned medium (10% v/v), Noggin conditioned medium (10% v/v) or recombinant protein, EGF, gastrin, FGF-10, nicotinamide and A83-01	PDOs culture was successful in 75% (3/4) and 83% (5/6) samples in Netherlands and USA respectively Human and murine pancreatic PDOs with molecular and cellular properties to evaluate neoplastic progression	[66]
Pancreatic cancer	159 samples from primary tumors and 138 metastases from surgical resections, fine-needle biopsies or rapid rapids autopsies	*Digestion:* Collagenase XI, DNAse and Y-27632 for 1 h at 37 °C *Culture:* Matrigel plus DMEM/F12 supplemented with HEPES, Glutamax, A83-01, hEGF, mNoggin, hFGF-10, hGastrin I, n-acetylcysteine, nicotinamide, PGE2 1, B27, Rspo-1 conditioned media 10% and afamin/Wnt3A conditioned media 50% *Drug screening:* 500 cells seeded in 10% of Matrigel. At following day were treated with 5 chemotherapeutics agents. At 6 days were measure viability using CellTiter-Glo	PDOs (n = 66) with efficiency of generation of 75% and 78% harbored genetic alterations and transcriptomic analysis revealed unique cluster consistent with PDAC PDOs exhibited heterogenous responses to chemotherapy and other target agents. In a single-cell case study, the retrospective clinical data was equal with the PDO chemosensitivity profile	[44]
Pancreatic cancer	30 patient-derived organoids from pancreas and distal bile duct tumor	*Digestion:* Collagenase incubate at 37 ºC *Culture:* Cultrex growth factor reduced BME type 2 plus medium supplemented with Wnt3a-conditioned medium (50% v/v), B27, n-acetylcysteine, nicotinamide, A83-01, FGF-10 and Noggin *Drug Screening:* PDOs were dissociated in single cells and cultured in 384-well, 24 h later drug was added. After 72 h was evaluated the viability of the cells using CellTiter-Glo	PDOs Biobank with 30 samples and retain histological features from tissue which derived and present the genetic alterations common in this type of tumor. In drug assay identify 76 compounds could be target of therapies in pancreatic tumor. 1 PDOs present similar resistance to gemcitabine compare with the clinical response	[67]
Pancreatic cancer	Biopsied tissue from patient enrolled in Clinical Trial: NCT03563248	*Digestion:* mechanical digestion and enzymatic digestion *Culture medium:* Matrigel plus Human complete feeding medium: DMEM/F12, HEPES Glutamax, A83-01, hEGF, mNoggin, hFGF-10, hGastrin I, n-acetylcysteine, nicotinamide, PGE2 1 μM, B27, Rspo-1 conditioned media 10%, Afamin/Wnt3A conditions media 50% *Drug assay:* Gemcitabine, paclitaxel, 5FU and oxaliplatin were tested in logarithmically curve. At 5 days, the viability was evaluated using CellTiter Glo	PDOs culture was successful in 77% (59/77). Tissue derived from biopsy the establishment was 78% (34/45) and samples from resection was 75% (24/32). The results demonstrate that the protocol reported could facility precision medicine in the treatment of pancreatic cancer	[43]
Pancreatic cancer	Biopsy tissue	*Digestion:* STEMxyme for 30–40 min. Then, Accutase for 30 min *Culture:* 5% Matrigel plus Y-27632 plus DMEM supplemented with growth factors, insulin and FGF-2 *Drug screening:* PDOs were dissociated to single cells and diluted to 25.000 cells/mL and cultured for 4 days. Then the drug was added. At days, cell growth was measured using Cyto Tox-Glo	PDOs culture was successful in 41% (31 of 75). In 12 PDOs were tested drug, showed sensitives to Gemcitabine, 5FU, oxaliplatin, SN-38 and paclitaxel Reported a strong indication that drug sensitives in PDO models have the capacity to predict clinical response	[51]
Pancreatic cancer	94 patients with PDAC, 43 from biopsy and 73 from surgical specimen	*Digestion:* Collagenase XI, DNAse I and Y-27632 in Human complete medium *Culture:* Matrigel plus Human Complete Medium: advanced DMEM/F12, HEPES, Glutamax, Primocin, A83-01, hEGF, mNoggin, hFGF-10, hGastrin I, n-acetylcysteine, nicotinamide, B27, 10% Rspon-1 conditioned media, 50% Afamin/Wnt3A conditional media *Drug Screening:* PDOS were dissociated in single cells, the drug evaluated were Gemcitabine, placitaxel, SN-38, and 5-FU and oxaplatin, cell viability was measure with CellTiter Glo	Generation a biobank with a total of 117 patients with different ethnicity. PDOs culture was successful > 70% and feasible from tissue originating from biopsy or surgical resection	[54]
Pancreatic cancer	Tissue from surgical resected PDAC Clinical trial (NCT03563248)	*Digestion:* culture medium and collagenase XI *Culture:* 5% Matrigel plus Y-27632 plus DMEM supplemented with growth factors, insulin and FGF-2 *Drug Screening:* PDOs were dissociated in single cells and plated on 384-well with 10% of Matrigel, Gemcitabine, paclitaxel, irinotecan, 5FU and oxaliplatin were tested in logarithmically curve, cell viability was measured 5 days after drug treatment using CellTiter Glo	Demonstrate the capacity of rapidly establishment to PDOs from PDAC, in according to the clinical time predictive biomarkers of chemotherapeutic response. In addition, was the first prospective experience using PDOs	[68]
Gastric cancer	37 GC, 2 adjacent, 7 nontumor and 9 normal like specimens were collected by surgical resection, endoscopic biopsy and ascites puncture	PDOs were established as reported Bartfeld et al., 2015 [80] *Digestion:* PBS/EDTA containing 2 × TrypLe for 1 h at 37 °C *Culture:* Matrigel and DMEN/F12 medium supplemented with HEPES, Glutamax, B27, Gastrin I, n-acetylcysteine, EGF, FGF-10, Noggin, R-spo1, Afamin-Wnt-3A serum-free conditioned medium and A83-01	PDO culture was successful in 74.6% (44/49) PDOs maintained histopathological and molecular subtypes in comparison with their parental tissues	[70]
Gastric cancer	20 tissue samples were obtained from surgical resection	*Digestion:* Collagenase IX and Dispase II- *Culture:* Matrigel and DMEN/F12 medium supplemented with Wnt3A 50%, Rspo-1 10%, Noggin 10% (both as conditioned medium), B27, Nicotinamide, N2, n-acetylcysteine, hFGF-10; mEGF, Gastrin and A83-01 *Drug screening:* PDOs were seeded in Matrigel. At following day were treated with 5 chemotherapy drugs at 3-point and selected PDOs were treated with trastuzumab, Palbociclib and imatinib at 2-point. At 24-72 h the cell viability was assayed using Presto Blue Cell viability reagent	20 PDOs were established and shown to represent characteristics and altered pathway corresponding to primary tissue A differential response to chemotherapy was observed	[46]
Gastric cancer	10 human cancer fundus tissues and normal controls were collected from surgical resection	*Digestion:* Collagenase for *Clostridium histolyticum* for 30 min at 37 °C *Culture:* Matrigel and DMEN/F12 medium supplemented with HEPES, L-glutamine, Pen/Strep, N2, B27, n-acetylcysteine, nicotinamide, EGF, Noggin, R-spo1 conditioned media, Wnt conditioned media, FGF-10, Gastrin I, Y-27632, amphotericin B/gemtamicin and kanamycin *Drug screening:* organoids were treated with 3 chemotherapy drugs at 8 different concentrations (0–200 mmol/L) for 48 h. The organoids proliferation was measured by MTS assay	PDOs closely resembled the patient´s native tumor tissues Tumor PDOs exhibited differences in the response to drug treatment 1 tumor PDOs were highly responsive to drug treatment and were derived from patient with a near complete tumor response Orthotopic transplantation of PDOs resulted un engraftment and development of human adenocarcinoma	[72]
Gastric cancer	42 PDOs derived from 34 patient tissues obtained from surgical resection	PDOs were established as reported Barker et al., 2010 [116] and Bartfeld et al., 2015 [80] *Digestion:* collagenases, hyaluronidase and Y-27632 for 1 h at 37 °C *Culture:* Matrigel and standard gastric organoid medium containing advanced DMEM/F12 supplemented with GlutaMax, HEPES, P/S, 50% Wnt3a, 10% Rspo-1 conditioned medium, 10% Noggin conditioned medium, B27, EGF, FGF-10, n-acetylcysteine, Gastrin, A83-01, Y-27632 and Primocin *Drug screening:* 15–20.000 PDOs per mL were coated with 50% Matrigel. At following day were treated with 37 compounds, 7 point, three technical replicated. At 6 day the cell viability assayed using CellTiter-Glo 2.0	PDOs success rate > 50% 2–3 weeks and maintained molecular subtypes, capturing regional heterogeneity and subclonal architecture to the original tumors Morphology, transcriptome and genomic profiles remain long-term similarity than in vivo tumors	[47]
Gastric signet ring cell cancer	Tissue from 26 patients who underwent surgery	*Digestion:* advanced DMEM/F12 containing collagenases, hyaluronidase and Y-27632 *Culture:* advanced DMEM/F12, HEPES, Glutamax, Pen/Strep, B27 supplement, n-acetylcysteine, nicotinamide, Noggin, Wnt3a, Rspon-1, EGF, EGF2, FGF-10, A83-01, Y-27632 and Gastrin *Drug screening:* Organoids were dissociated, 5000 cells were evaluated a 6 concentration of 5-FU, oxaliplatin, irinotecan and docetaxel. Viability was measured with CellTiter Blue 72 h post treatment	Generation to biobank of GC organoids, evaluation to respond a therapy was completed in 4 weeks, indicating that the model could be treatment recommendations	[33]
Biliary tract carcinomas (BTC)	18 surgically resected tissue specimens obtained from BTC patients	*Digestion:* collagenase type XI and dispase type II for 1 h at 37 °C *Culture:* Matrigel and organoid medium containing advanced DMEM/ F12 supplemented with Glutamax, HEPES, Pen/Strep, N2, B27, EGF, n-Acetylcysteine, gastrin, nicotinamide, 10% Rspo1 conditioned medium, A83-01, Forskolin and Y-27632 *Drug screening:* 12,000 cells were plated and cultures for 4 days, and compounds were added al final concentration 0.1 mm. After 6 days, the cell viability was evaluated using water-soluble tetra- zolium salts assay	PDOs culture was successful in 50% (3/6) of IHCC and 20% (1/5) of GBC BTC-PDOs recapitulated the histopathology, gene expression and genetic alterations evident in the primary tumors SOX2 could be a potential prognosis biomarker for patients with BTC The antifungal drugs amorolfine and fenticonazole suppressed the growth of PDOs with minimal toxicity to normal epithelial cells	[39]
Gallbladder cancer	Surgically resected tumor tissues from 41 untreated GBC patients	*Digestion:* collagenase IV for 30–60 min at 37 °C *Culture:* Matrigel and organoid medium containing advanced DMEM/ F12 supplemented with B27, N2, n-acetylcysteine, Noggin, EGF, FGF-10, IGF, HGF, Gastrin, Y27632, nicotinamide, A83-01, Forskolin, dexamethasome, primocin, Pen/strep, Glutamax and HEPES *Drug screening:* 5000 cells were plated and the drug were added at final concentration of 10 µM then 24 h after cell seeding. At 4 days, the cell viability was evaluated each three days using CellTiter-Glo	PDOs culture was successful in 12.2% of GBC (5/41) PDOs recapitulates the histopathology, genetic, transcriptomic and intratumoral heterogeneity of the primary tissues PDO are a potentially a useful platform to explore molecular pathogenesis and discover personalized drugs	[49]

*B27* B-27 supplement (the standard for neuronal cell culture), *N2* N-2 supplement CTS (cell therapy systems), *EGF* epidermal growth factor, *Rspo-1* R-spondin 1, *PGE2* prostaglandin E2, *CT-guide* computed tomography-guide, *mCRC* metastatic colorectal cancer, *mGOC* metastatic gastroesophageal cancer, *mCC* metastatic cholangiocarcinoma, *PBS/EDTA* phosphate buffered saline (PBS) with EDTA, *TrypLe* DMEN/F12 Dulbecco’s Modified Eagle Medium/Ham’s F-12 basal media, *FGF-10* fibroblast growth factor 10, *Wnt-3a* recombinant human Wnt-3a protein, *HGF* human hepatocyte growth factor, *HEPES* (4-2-hydroxyethyl)-1-piperazineethanesulfonic acid, *FOLFOX* 5-FU, leucovorin, oxaliplatin, *FOLFIRI* folinic acid, fluorouracil and irinotecan, *RECIST* response evaluation criteria in solid tumors, *BME* basement membrane extract, *FGF-2* fibroblast growth factor 2, *EZH2* enhancer of zeste 2 polycomb repressive complex 2 subunit, *HCC* hepatocellular carcinoma; combined, *HCC/CC* hepatocellular carcinoma plus cholangiocarcinoma, *IHCC* intrahepatic cholangiocarcinoma, *GBC* gallbladder cancer, *IGF* insulin growth factor

Cancer GI-PDOs have been proposed as potential reliable preclinical platform that could fill the void between cancer genetics and patient trials. In the following sections, we will highlight chronologically the principal studies that support the translational use of specific GI cancer PDOs. Table 2 summarizes the sample precedence and methods used for PDO establishment from specific GI sources. Outcomes from these platforms in the study of tumor biology, heterogeneity and plasticity are also summarized.

### Colorectal cancer PDOs (CRC-PDOs)

The first report of human gastrointestinal PDO culture was in colon cancer [60]. Building upon previous experiences in murine small intestine [31], Sato and colleagues were able to establishment a robust protocol to obtain the long-term culture of primary human epithelial cells from small intestine, colon, adenocarcinomas and Barret´s esophagus. To establish the PDOs, colon cancer cells did not require the addition of multiple growth factors, however, EGF, nicotinamide, an inhibitor of sirtuin activity [61], an inhibitor of activin receptor-like kinase (ALK), 4/5/7 (A83-01), and a p38 inhibitor (SB202190) were added, which significantly improved the efficiency and prolonged culture time. Subsequently, Van de Wetering and colleagues [40] used culture media lacking Wnt, and further supplemented the culture with a selective ROCK inhibitor (Y27632) and prostaglandin (promoters of intestinal repair). This preparation generated organoids from resected colorectal cancers with close to a 90% success rate, recapitulating their respective molecular subtype. Furthermore, they were the first research group to coin the term “*living biobank*” to refer to an organoid biobank. Through whole-exome sequencing (WES), these researchers demonstrated that patient genomic alterations were maintained in vitro*,* and exhibited driver mutations and the hypermutated and non-hypermutated mutational profiles associated with CRC. Furthermore, a 3D drug screening proof-of-concept showed high-quality reproducibility between CRC organoids (n = 18) with their genomic features and drug sensitivity, favoring the identification of useful genetic markers to predict drug resistance in CRC patients (for example *KRAS* and *TP53* wild-type organoids were sensitive to cetuximab and Nutlin-3a [40]. Using the same Sato protocol, in 2015 Weeber and colleagues [22] were able to establish CRC-PDOs from biopsies of colon cancer metastases with high preservation of somatic mutations and DNA copy number profiles [22]. Using slightly different protocols, four subsequent studies showed that CRC-PDOs could be established with a success rate of 70 to 100% [37, 38, 52, 62]. The subsequently study by the Sato group [62] successfully established organoids with 100% efficiency from varying and rare histological sub-types, such as poorly differentiated adenocarcinoma and neuroendocrine carcinomas. Moreover, they found that niche factor independent growth was associated with adenoma-carcinoma transition and accumulation of multiples mutations [62]. A recently reported biobank containing 50 CRC organoids derived from primary tumors and paired liver metastatic lesions (CRLM) confirmed that: (i) organoids maintain the histopathological identity of the corresponding tumors of origin, (ii) recreate the different consensus molecular subtypes (CMS) of CRC, being CMS2 and CMS4 the most represented and (iii) show wide degrees of intra and intertumoral heterogeneity [37]. Interestingly, the authors also reported that CRLM-PDOs could predict the chemotherapeutic response to both FOLFOX and FOLFIRI regimens, correlating the in vitro sensitivity to both antitumor regimens with patient progression-free survival [37]. Finally, a network-based machine-learning (ML) method [53] examined pharmacogenomic data from a biobank of CRC-PDOs [40] revealing that components of the “activation of BH3-only proteins” pathway were associated with high chemosensitivity to 5-FU in CRC-PDOs. Subsequently, this pathway was validated as predictive biomarker of therapeutic efficacy and overall survival in a cohort of 114 CRC patients (data obtained from TCGA; https://www.cancer.gov/tcga), with the patients sensitive to 5-FU having a higher overall survival compared to resistant patients. This study highlights the importance of generating large-scale biobank-derived data from PDOs, and that when combined with ML methodology, an improvement in the predictive ability of drug-response for cancer patients can be achieved. Two principal research groups have successfully established rectal organoids from tissues obtained by biopsy forceps, with an overall success rate of 77–85.7%. In 2019, Ganesh and colleagues set out to derive PDOs from patients with either primary, metastatic or recurrent disease from resected or biopsied rectal cancers, while a year later, Yao and colleagues focused on naïve locally advanced rectal cancers [63]. Both groups demonstrated that rectal PDOs recapitulated the pathophysiology and genomic profiles of corresponding primary tumors [48, 63]. Response curves for the chemotherapies 5-FU, FOLFOX and irinotecan and radiotherapy curves supported the use of PDOs to mirror clinical response.

### Liver and pancreatic (PDAC) PDOs

The first efforts to establish liver PDOs were performed by the Huch laboratory, who modified the protocol for growing normal liver organoids to favor propagation of primary liver cancers from the resection of three cancer subtypes: hepatocellular carcinoma (HCC), cholangiocarcinoma (CCA) and combinate phenotype HCC/CCA [41]. In a subsequent study, the HCC and CCA-PDOs were established from needle biopsies with 33% (10/38) success rate, and these could be maintained in culture for up to 32 weeks [64]. In both studies the human HCC PDOs were able to recapitulate the primary cancer*,* showed tumorigenic and metastatic potential and facilitated the prediction of drug response [41]. Interestingly, the drug screening testing in liver-PDOs was able to identify the ERK inhibitor CH772984 as a potential therapeutic agent and test sensitivity to sorafenib [41, 64].

The first developed methods to generate and propagate human pancreatic cancer PDOs (PC-PDOs) was reported by Boj and colleagues based on the optimization of a previous approach using healthy adult murine pancreas ductal cells [65]. Using slightly different protocols, eight subsequent studies showed that PDAC-PDOs can be established with success rates of 42 to 90% [42–44, 51, 54, 66–68]. In each study the PDOs were able to recapitulate the histology and contain the genetic alterations of the original tumor. Recently, a biobank of PDAC-PDOs collected tumor tissue before and after donor patients underwent neoadjuvant chemotherapy (NAT) based in the cytotoxic agents Gemcitabine, Paclitaxel and FOLFIRINOX (FFX) [54]. The authors determined that PDAC-PDOs exhibit differential sensitivity to each drug and PDOs derived from pre-NAT (100%) and post-NAT (71%) were able to predict the clinically observed response against oxaliplatin in patients treated with FFX, which is consistent with previous reports in pancreatic cancer [44]. Taken together, PC-PDOs were able to reflect human pancreatic ductal cells, represented a transplantable model of human pancreatic cancer progression, and were employed for high-throughput screening. Furthermore, these results support the potential use of PDOs as a preclinical tool to decide possible PDAC therapeutic strategies as well as to study changes in drug sensitivity during disease progression. However, the main challenge using PDAC PDOs is try to replicate the stromal components present in the TME (mainly fibroblast and macrophages), which is fundamental to achieve a representative model of the pathophysiology that underlies this cancer type [69]. Several approaches aimed at improving this interaction have been use of co-culture, minitumors or the tumor on a chip-platforms.

### Gastric PDOs (GC-PDOs)

Six independent groups have reported the generation of GC-PDOs [33, 47, 52, 70–72]. The overall success rate has been between 50–75%, and as seen with other PDOs, the GC-PDOs maintained histopathological, molecular subtype and pathway alterations in accordance with their parental tissues. Phenotypic analysis of GC-PDOs have shown that divergent genetic and epigenetic routes gain Wnt and R-spondin niche independence and the mutational spectrum of different organoids matched previously identified molecular GC sub-type [46, 70]. Furthermore, the mutational landscape offered targeted therapy with trastuzumab for ERBB2 alteration and palbociclib for CDKN2A loss [46]. Yan and colleagues in 46 molecularly characterized organoids [47] emulated the pathophysiology of the four different molecular subtypes of gastric cancer, including Epstein-Barr virus (EBV), microsatellite Instability (MSI), intestinal/chromosome instability (CIN) and diffuse/genomically stable (GS) and measured drug response curves and revealed that some chemoresistance GC-PDOs showed sensibility to alternative clinically approved drugs [47]. PDOs have also been established from signet ring positive gastric cancer (SRCC) (n = 4), and non-SRCC (n = 8) subtypes, with both tumor subtypes maintaining characteristics of parental tissue, including GC-related markers (pan-CK, CEA), histological architecture and frequently mutated genes in GC (TP53, TTN, and CSMD1). Accordingly, divergent and heterogeneous chemosensitivities were observed between SRCC and non-SRCC organoid cultures, with the SRCC-PDOs being more sensitive to docetaxel than non-SRCC organoids. In 2022, Kumar and colleagues performed a comparative cell-state analysis between four pairs of GC-PDOs and their primary tumors by single cell-seq data [73]. Interestingly, with the exception of a depletion in lymphoid and plasma lineage, the GC-PDOs were able to recapitulate the five major cell types. Furthermore, both healthy tissue and tumor-derived gastric PDOs showed expression of epithelial cell-related genes involved in differentiation/dedifferentiation associated with increased transcriptional plasticity [73, 74].

### Biliary tract cancer PDOs (BTC-PDOs) and Gallbladder cancer PDOs (GBC-PDOs)

The first establishment of intrahepatic cholangiocarcinoma (IHCC) PDOs was reported by Broutier and colleagues in 2017 [41]. Later, PDOs were successful established from additional cases of IHCC and gallbladder cancer (GBC) with a success rate of 50% and 20% respectively. In 2019, Yuan and colleagues reported the establishment of five GBC PDOs with a success rate of 12.2% (5/41) and reported their ability to maintain stability in culture for more than 6 months. The BTC-PDOs closely recapitulated the histopathological, genetic alterations and gene expression features of the primary tumors [39, 49]. Interestingly, GBC PDOs also maintained the intratumoral cellular heterogeneity of their derived tissue at the single-cell level [49]. Using a FDA drug screening approach, applying the dual PI3K/HDAC inhibitor CUCDC-907 and two antifungal drugs, a significant reduction in growth was shown in various tumoral BTC-PDOs with minimal toxicity observed in organoids derived from corresponding healthy tissue [39, 49]. The low rate of success in BTC-PDOs may be due to either a lack of long-term expansion or the presence of contaminating non-cancerous cells (removed during surgical resection) that then expand and outcompete the cancer cells [49]. Aspects that need to be addressed to increase PDOs formation efficiency are the incorporation of good quality samples (high tumoral cellularity) and the optimization of protocols.

## Gastrointestinal patient-derived organoids as part of a representative tumor microenvironment (TME) to allow therapeutic prediction

Cancer biomedicine is focused not only in understanding the biological differences that underlie each tumor type, but also the differences between the individual patient, even when grouped by their pathological behavior. To this end, the analysis of the TME has been increasingly put in perspective, with promising discoveries. The implementation of PDO technology in cancer research has been crucial to enhance the development of personalized or precision medicine, but given its exclusively epithelial origin, it lacks other cell types that belong to the TME, limiting its ability to faithfully simulate the structural and physiological components of the bulk tumor. Thus, the establishment of cellular co-cultures of organoids enriched with a specific cell (mainly pathogens, stromal, inflammatory and immune cells), is a method that offers a solution to obtain a model that more closely resembles the complexity of the TME. This will also allow an approximation as to the specific patient pathology profile, which will aid understanding of the TME-tumor cell interaction and the subsequent impact this has on tumor progression. This objective is to bring the PDO model closer to the goal of predicting patient treatment response [16, 75].

Gastrointestinal organoids co-cultures have shown utility in deciphering the underlying mechanisms involved in carcinogenesis promoted by oncogenic pathogens such as cancer-associated bacterium [76]. For instance, a widely studied pathology has been the chronic infection caused by *Helicobacter pylori (H. pylori)* that affects around 50% of the world population, of which a subgroup of patients can progress to gastric cancer [77]. After a *H. pylori* infection, gastric organoids have shown both increased proliferation and activation of stem cells associated with oncogenic virulence factor CagA [78, 79]. Organoids infected with *H. pylori* demonstrated signs of inflammatory response as seen by NF-kB pathway activation and overexpression of IL-8 [80], Furthermore, a significant increase of PD-L1 expression was observed in these organoids [56]. Similar to this inflammation-related cancer model, the genotoxic effect of *Salmonella* infection has been proposed as an “inflammatory stimulus” that induces malignant transformation of the gallbladder epithelium [81]. In genetically predisposed murine gallbladder organoids, *Salmonella typhi* (*S. Typhi*) infection has been associated with TP53 mutations and c-MYC amplifications [82]. Furthermore, in human gallbladder organoids infected with *S. Typhi*, the activity of typhoid toxin (CdtB subunit) brought about cell cycle arrest and DNA damage, which was extended to non-infected cells by a paracrine effect [83]. Further studies have used intestinal organoids to elucidate the pathogenesis of gastrointestinal disorders caused by host–pathogen interaction. For example, intestinal organoids have been used to understand the pathogenic role of *Clostridium difficile*, a commensal bacterium which has the ability to disrupt epithelium barrier function [84]. Recently small intestinal organoids co-cultures with *Lactobacillus casei* and *Bifidobacterium longum* growing under anaerobic condition have demonstrated a probiotic effect, together with improving barrier formation and mucin regulation [85]. In the clinical setting, co-culture models have also promised prediction of therapeutic efficacy in patients with gastrointestinal neoplasms such as locally advanced rectal cancer [86] and CRC [87]. In these assays, interaction of tumor organoids with immune cells, including T lymphocytes, macrophages-associated cancer (MAF), among others, were investigated. Nevertheless, one of the main challenges now is to recreate the tumor-associated stroma and introduce vasculature (blood vessels) into this model. As technology advances, the aim will be to maintain differentiated cancer cells, stem cells, pathogens and endothelial cells together and with interaction on a 3D substrate matrix [88]. This strategy is novel and obtaining a vascularized organoid greatly broadens the perspectives and applications in drug delivery study. Designing and implementing a standardized protocol for obtaining organoid culture with each pathophysiological component of the TME, will creating a more reliable model to compete, and one day leave obsolete, the use of animal models in drug safety and screening.

### Gastrointestinal PDOs and fibroblast interaction

Cancer associated fibroblasts (CAFs) contribute to carcinogenesis and tumor development through the production and delivery of growth factors, cytokines, pro-angiogenic factors and extracellular matrix (ECM); all of which have been related with the hallmarks of cancer and therapy response [89–91]. Promising results are starting to emerge using GI-PDOs in co-culture with CAFs. The first report to demonstrate cooperative interaction between PDOs and CAFs was reported by Öhlund and colleagues in 2017 [92]. The main focus of this report was the elucidation of the desmoplastic reaction associated with stellate cell activation (the major source of CAFs in pancreatic ductal adenocarcinoma) [92]. In this study, using two different co-culture approaches, the authors were able to identify two CAF subtypes present from patient samples. Through the direct co-culture in Matrigel of PDAC-PDOs with pancreatic stellate cells (PSCs), the authors recapitulated the desmoplastic reaction in vitro*,* with PSCs converting from a resting quiescent state to a stroma-producing CAF (called myofibroblast CAFs, myCAF), which was characterized by elevated expression of ⍺-SMA and high collagen I deposit in the co-culture [92]. Utilizing conditioned media from human PDAC-CAFs (CAFs growing on a transwell system) with patient-matched tumor organoids seeded in Matrigel, the authors tested the paracrine interaction and described another distinct subpopulation of CAFs (called inflammatory CAFs, iCAF), which were characterized by lack of ⍺-SMA, induction of secrete IL-6 and activation of STAT3 in PDAC-PDOs [92]. Subsequently, utilizing the same transwell co-culture system but now using conditioned medium from PDAC-PDOs grown on the transwell insert and CAF in the lower chamber, showed that soluble factors produced from tumoral cells are capable of inducing both previously reported myCAFs and iCAFs, which were differentiated by the expression of classical markers ⍺-SMA and IL-6, respectively [93]. Through this approach it was possible to elucidate the molecular components (secreted by tumor cells) which were responsible for the generation of these two CAF subpopulations. IL-1 signaling was the main pathway responsible for the induction of the iCAFs, while TGF-β induced the myCAF phenotype. Interestingly, the transplant of PDOs and CAFs into mice model showed a differential spatial distribution, with the myCAFs surrounding the tumoral cells while iCAFs were located away from the tumor cells in the dense stromal tissue. This suggested to the authors that the spatial distribution of CAFs have an impact in their functionality [93]. Taken together, these observations using PDAC-PDOs and CAFs co-culture highlight the power of this platform as a tool to study the plasticity of CAFs populations, understand their role in PDAC progression and may allow the analysis of new therapeutics directed at each population.

Subsequently, a similar approach was used by Liu and colleagues in 2021 [94] to research the role of CAFs in liver PDOs. The contact with liver PDO and CAFs in paracrine co-culture (through transwell culture system) showed that CAFs promoted the organoid growth and development. Interestingly, the direct liver cancer PDOs-CAF co-culture increased PDOs size and ki-67 expression, and the up-regulation of stem cell markers CD133, NANOG and TERT [94]. Transplantation of LC-PDOs with CAFs into immunodeficient mice observed more efficient tumor growth in xenograph model as compared to PDOs alone. Moreover, they showed that the presence of CAF or conditioned medium of CAFs conferred liver cancer PDOs resistance to anticancer drugs such as sorafenib, regorafenib and 5-FU [94].

**Fig. 2:**
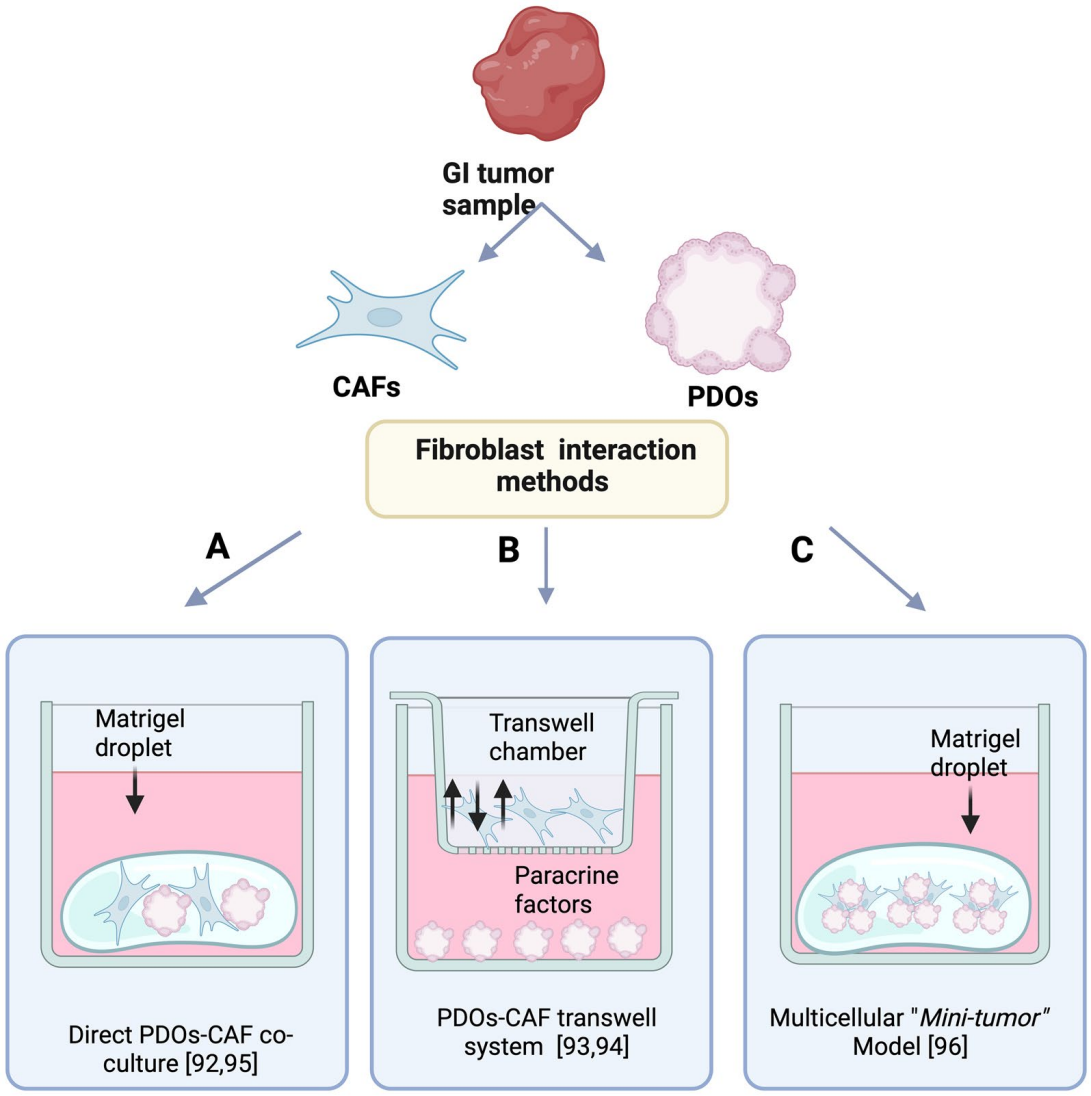
The main models to mantain the interaction between gastrointestinal cancers PDOs and CAF cells. **A** Direct PDOs and CAFs co-culture model using the Matrigel droplet method to emulate the ECM. **B** Model is a PDOs-CAF transwell system, which is used mainly for the evaluation of paracrine communication co-culture interaction between PDOs and CAF. **C** “Mini-tumor” co-culture, this approach has a previous step allowing the preincubation of CAFs-PDOs in low attachment plates to improve the interaction, before co-culture is achieved using the Matrigel droplet method. The figure was created with Biorender

Considering that the CRC extracellular matrix is rich in hyaluronan and collagen, in 2021 a new approach was formulated using CRCPDOs encapsulated within a 3D hyaluronan-gelatin hydrogel with subsequent co-culture of patient-derived CAFs [95]. Using this approach, the authors showed that in PDOs cultured in absence of growth factors, the addition of the CAFs maintained the proliferation of PDOs and restored the biological pathways absent in the PDO culture alone. These pathways were identified as the ECM-receptor interaction, focal adhesion, chemokine and PI3K-Akt signaling pathways, which have been previously associated with cancer-CAF crosstalk and are typically altered in tumor tissues [89]. This model also showed promise in evaluating antineoplastic drugs, offering yet again a tool for potentially personalized medicine evaluation. Subsequently, Harryvan and colleagues [96] established a novel human multicellular “mini-tumor” model containing PDAC-PDOs and patient-derived CAFs that, in comparison with the abovementioned colorectal model, recapitulated both desmoplastic and differentiation of PDAC in the myCAF subset. In this approach, the CAFs and PDOs where previously pre-incubated in ultra-low attachment plates to achieve an interaction between both cell types before the “mini-tumors” where seeded into Matrigel. Using this novel model, the authors showed that PDOs-CAF “mini-tumors” were able to secret collagen type I, generating an ECM surrounding de tumor cells and induce Epithelial to Mesenchymal (EMT) programing, reflecting how this interaction between the parenchyma tumoral and its stroma impact tumor progression. Finally, the authors reported that CAFs confer oxaliplatin resistance to PDAC-PDOs, again suggesting these models as high throughput screening platforms. In the Fig. 2, we summarize the tested methods commonly used for PDO and CAF co-cultures. However, the selection of the optimal co-culture method will depend on the specific research question that needs to be addressed. For example, if the effect of the physical contact between PDOs and CAFs is under question then necessary the direct interaction between both cells is required, however, to evaluate the effect of paracrine secreted signals a transwell co-culture system is better suited. To explore both physical and paracrine aspects, the use of “mini-tumors” represent a better complexity option to study stroma-dense tumors as pancreatic cancer [97]. This in vitro PDO-CAF direct approach is shown to promote both the desmoplastic reaction and differentiation toward the myCAF (including myCAF and iCAF), which can more faithfully simulate the pathological features of original tumor. Examples of this may be the genetic and mesenchymal characteristics, spatial organization of the TME, the level of sensitivity to anticancer treatments and CAF heterogeneity. Certainly, the physical and physiological integration between stromal and tumor epithelial cells provided by “mini-tumors” recreates more faithfully the heterogeneity of the primary tumor, highlighting its usefulness as a preclinical model. The future challenge is to continue improving these models to include additional stromal cell types and extend this model to study GIs cancers not currently tested. The novel biological questions that can be answered using these models maybe the examination of the rigidity (stiffness) of the Matrigel and how this impacts on the paracrine crosstalk [98]. This may also lead to the enhanced design of Matrigel or ECM substitutes to obtain the optimal tumor ex vivo microenvironment.

### Gastrointestinal patient-derived organoids as a system to immunotherapy prediction

The evaluation of interactions between organoids and immune cells would make it possible to explore new cancer treatments, and this process may be as equally applicable to immunotherapy. Since the initial approval of immunotherapy for the treatment of melanoma, the use of different checkpoint inhibitors has revolutionized the immunotherapy treatment in other solid tumors. Immunotherapy has now become a standard of care in many cancer settings during the last decade with the introduction of specific antibodies blocking immune checkpoint molecules, such as cytotoxic T-lymphocyte-associated protein 4 (CTLA4) and programed death 1/programed death-ligand 1 (PD-1/PD-L1) among other burgeoning immune checkpoint pathway inhibitors arriving to the clinic [99]. In gastrointestinal cancers, the FDA has approved the immunotherapy drugs Pembrolizumab (a PD-1 humanized IgG4 monoclonal antibody) as first-line treatment in combination with trastuzumab and chemotherapy in patient with locally advanced unresectable or HER2 positive gastric cancer or gastroesophageal junction carcinoma [100] or for MSI-H or mismatch-repair-deficient (dMMR) advanced unresectable or metastatic colorectal cancer tumors [101]. Moreover, FDA approved nivolumab (PD-1, monoclonal antibody) plus chemotherapy for advanced gastric, gastro-esophageal and esophageal adenocarcinoma [102].

**Fig. 3:**
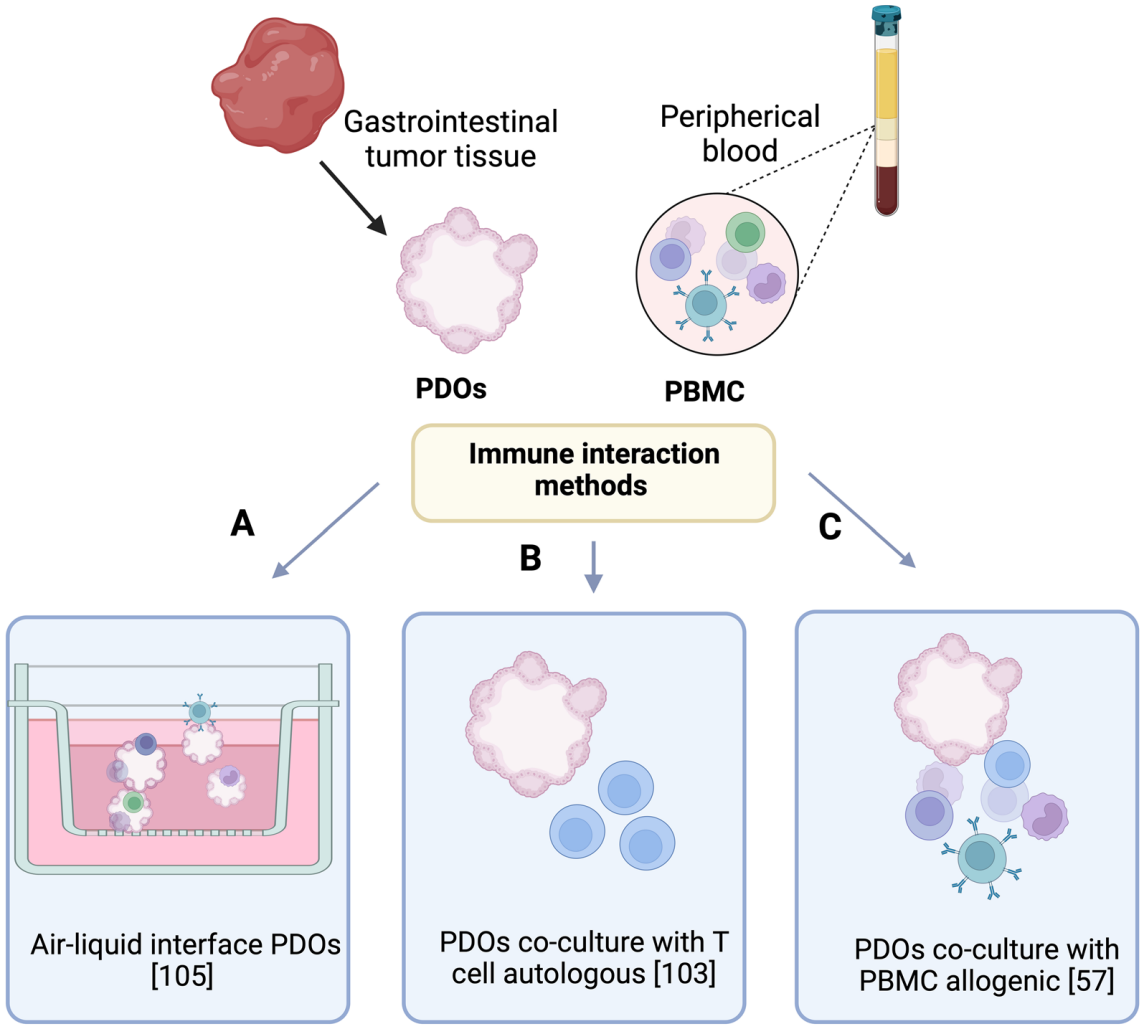
The main models to mantain the interaction between gastrointestinal cancers PDOs and immune cells. **A** Model based in the use of ALI-PDOs that preserve the immune cells for almost 60 days. **B** Co-culture interaction between PDOs and T-cells derived form same patient (autologous) with the aim to generate tumor-responsive as a cancer treatment. **C** Co-culture interaction based on PDOs and PBMC derived from healthy controls (allogenic) with the aim to generate cytotoxicity activity against to the tumor cells from CD8 T-cells. The figure was created with Biorender

Given this context, different research groups have worked on improving organoid complexity by adding niche/immune cells, with the aim of predicting in vitro the immunotherapy response in patients (Table 3). In the Fig. 3, we summarize the tested methods commonly used for PDO and immune co-culture. The first proof of concept that tumor organoids can be used as ex vivo platform to study T-cell interaction for individual patients was reported by Dijkstra and colleagues in 2018 [103]. The co-culture model established by Dijkstra and collaborators [103] was derived from mismatch repair-deficient colorectal cancer (dMMR CRC) patients and non-small-cell lung cancer patients. Both type of organoids interacted with autologous peripheral blood mononuclear cells (PBMC) isolated from the same patient. This co-culture model demonstrated that 50% of patients with dMMR CRC were MHC-class competent and able to induce CD8T cell activation. In this study the tumor recognition by CD8 + T was evaluated after 2 weeks of co-culture between organoids and T-cells by evaluating organoid-induced IFN-γ secretion and upregulated expression of CD107a, a marker of degranulation of cytotoxic NK and CD8 T-cells. In addition, the authors reported the induction of PD-L1 in IFN-γ pre-stimulated organoids as a control. Evaluating the effects of PD-L1 inhibitors in this setting also highlights the effort to further evaluate the impact of immune checkpoint blockage using this platform. In conclusion, the co-culture of autologous tumor organoids with PBMC can be used as a strategy to assess the tumor sensibility to T-cell-mediated attack and thus predicted the patient response to immunotherapy [103, 104]. However, there is a necessity to extend this evaluation to other poorly immunogenic tumors. Furthermore, strategies are required to avoid or revert MHC I loss, which is a mechanism the cancer cell utilizes for immune escape.

**Table 3:** Principal studies reporting GI cancers PDOs in co-culture with immune cells

Type of organoids	Immune cells	Aims Co-culture	Results	References
Colorectal tumor organoids	Lymphocyte from Peripheral blood	Analysis of specific-tumor T cells response against cancer cells in a personalized profile	Generation of co-culture between tumor cells and autologous PBMC. Generation of TCD8 reactive against tumor cells	[103]
Colorectal cancer organoids	Development new protocol to organoids from tissue airface that retain the components present into to the tumor for a day	Generation of organoids 2.0 which retain microenvironment of the tumor included T cells	Preservation of tumor microenvironment, but limited time for use	[105]
Mouse intestinal organoids	Intraepithelial Lymphocyte (IELs)	Evaluating the interaction between intestinal epithelial cells and intraepithelial lymphocyte	Generation of co-culture in presence of cytokines. Multi-directional movement to IELs along of surface present in organoids	[117]
Gastric organoids	Dendritic cells and T cells from peripheral blood	Evaluate the interaction between cancer cells infected with *H. pylori* and immune cells	Organoids infected with *H. pylori* expressed PD-L1 immune checkpoint and suppressed the T cells response	[56]
Mouse gastric organoids	Dendritic cells and T cells	Evaluate the role of Hh signaling in PD-L1 expression Understand the feasibility such as a model to predicted immunotherapy response in gastric cancer	Treatment with anti-PD-1 induced apoptosis in tumor cells Generation of murine models of co-culture organoids-immune cells	[118]
Pancreatic cancer organoids	Multy-cells type (fibroblast and T cells)	Generate and characterized the models of co-culture and demonstrated the applicability in pancreatic cancer	Demonstrated a tumor microenvironment generated by the co-culture of pancreatic organoids with stroma and immune cells	[119]
Cholangiocarcinoma cancer organoids	PBMC or T cells (Healthy control)	Establish and optimize a 3D co-culture model for study the growth, inhibition of organoid cell death by cytotoxic activity of T cells	The results demonstrated the different response between patients, and the capacity to the T-cells to generate cytotoxicity activity against the organoids	[57]

*PBMC* peripheral blood mononuclear cells, *TCD8* T cells type CD8, *PD1* programmed cell death protein 1, *PD-L1* programmed death-ligand 1

The reconstitution of organoids with immune cells as air–liquid interface patient derived organoids (ALI-PDOs), also named “organoids 2.0”, is a promising model in biomedical research [105]. The organoid 2.0 strategy, founded in the ALI-PDOS generated by Neal and colleagues [105], is a model based in patient-derived organoids of tumor epithelia that retain native immune cells and non-immune stromal cells, recapitulating the TME diversity [16]. Briefly, the authors utilized WNT3A, EGF, NOGGIN, and RSPO1 (WENR) supplemented medium and mechanically processed tumor fragment growing in air–liquid interface to establish PDOs from 100 individual patients with different subtype and localization of cancers, including 20 colorectal cancers, 11 pancreas, 20 kidney, 20 lung and 29 other tumor types. These organoids could be maintained in long term culture and were able to preserve the original immune cells such as cytotoxic T cells, T helper cells, B cells, Natural Killer (NK) cells and macrophages. Furthermore, the tumor-infiltrating lymphocytes (TILs) were maintained functionally active and kept the relative expression of PD-1 protein between fresh tumors and organoids. The stroma of this PDOS contained myofibroblasts closely associated with epithelium, decreasing immune cells and fibroblast stromal cells over a period of 1–2 weeks month [105]. Moreover, to demonstrate TIL functionality, PDOs were treated with Nivolumab and the response rates at 7 days were sufficient to elicit tumor cytotoxicity. Interestingly, these in vitro activities were concordant with the clinical response in respective patients. However, the next challenge is to increase the clinical applicability of this model. The culture model needs to improved, as currently the immune cells present in ALI-PDOS are not supported beyond 60 days and future prospective studies are required to correlate PDOS and patient immunotherapy response [105].

In 2022, studies showed the feasibility of developing co-culture organoids from cholangiocarcinoma (CCA) with allogenic PBMCs [57]. This “proof-of-concept” generates a model to evaluate the response of organoid-immune cell co-culture with the perspective of predicting the response to immunotherapy. The authors described the optimal conditions to generate the co-cultures which include the use of Matrigel or similar matrix, for example, Base Matrix Extracellular (BME) at concentration of 10% to maintain organoid 3D morphology and allow PBMC interaction with the tumor cells [57]. A noteworthy aspect of the investigation carried out by Dijkstra and his group is the use of allogenic conditions in the co-culture. The model used PBMCs from healthy donors with MHC mismatch, which circumvented the necessity to use specific (autologous) patient blood in maintaining the capacity of immune cells to attack the tumor cells [57]. This proof of concept is a first step for the future use of a clinically relevant co-culture model in precision medicine.

The maintenance of interaction of PDOs with other cells of the TME is relevant to understand the behavior of tumor cells, the progression of cancer and the understanding of the mechanisms for drug resistance and/or evasion of immune response. Despite varying studies showing the potential and feasibility of organoid and immune cell co-culture, further studies and large patient number validations are required to arrive at a clinically relevant tool in personalized medicine.

## PDOs as a tool to predict the therapeutic response in GIs patients

In the previous sections we discussed the use of incorporating aspects of the TME in PDO models for prediction of immunotherapy response, however the use of chemotherapy is still a mainstay of GI tract cancer treatment and the response to this treatment has been the subject of examination in the PDO system. Despite over 40% of patients responding to the standard first line regimens, currently there are no good biomarkers or tests guiding the efficacy of chemotherapy [47]. Despite considerable efforts to maximize drug efficiency and minimize the side effects of chemotherapy, the ability to anticipate patient response and tumor regression is still elusive, leading to a dangerous loss in time which allows cancer progression and an unnecessary loss of quality of life for the patient. The quantity of publications referring to response prediction in in GI-PDOs have greatly increased in the last five years. In nine original manuscripts reviewed, the authors correlated drug response with certain parameters of clinical response to treatment in GI-PDOs [37, 38, 44, 47, 51, 52, 63, 68, 72].

In a study by Tiriac and colleagues, chemotherapy addition delivered heterogeneous responses in PDAC-PDOs, although in specific cases they did parallel patient outcomes. The authors concluded that with PDOs, a combination of molecular and therapeutic profiling may predict patient response and help in the selection of antineoplastic treatments [44]. Recently Seppala and colleagues isolated PDAC-PDOs prior to pharmacotyping and treatment with FOLFIRINOX (FFX) and similarly showed preliminary evidence for prediction of therapeutic response in a subset of the patients. In this study, PDO pharmacotypes suggested sensitivity to FFX and accordingly favorable RECIST conclusions were observed together with reductions in the tumor marker CA-19–9 [68]. The Harnessing Organoids for Personalized Therapy (HOPE trial) was a pilot prospectively trail that tested the feasibility of generating PDOs from PDAC and correlating chemotherapy combinations by drug response curves to clinical response by RECIST. Drug testing was performed on 12 organoids, with eight organoids sequenced and nine sent for RNA-Seq analysis. Dose–response curves and area under curve (AUC) for multiple drug combinations ranged from 0.25 (highly sensitive) to 1.0 (resistant), however the authors concluded that their findings showed promise of utilizing PDOs to increase drug response rates and minimize toxicity by ruling out ineffective treatments [51]. In other studies using gastric cancer PDOs, which incorporated a small sample size, one case was present where the PDO was highly responsive to drug treatment, and similarly the patient’s tumor exhibited a near complete response using the same chemotherapy combination therapy [72]. Another study in GC-PDOs extended this latter observation to three additional patients [47]. Furthermore, a recent study using liver metastasis from primary colorectal cancer (CRLM) demonstrated that CRLM-PDOs (n = 13) showed predictive power in the response to FOLFOX or FOLFIRI, correlating the response by RECIST and the progression-free survival of the donating patients [37].

At the moment there are three highly relevant studies that have presented GI-PDOs as predictors of anti-neoplastic drug and radiotherapy response [38, 48, 52]. Vlachogiannis and colleagues established PDOs from sequential biopsies of liver metastasis from gastroesophageal cancer patients and examined their clinical predictive value in 21 comparisons between PDOs and clinical response. Interestingly, the authors reported that when compared with the clinical response in patients, the ex vivo cell viability response to chemotherapy and targeted therapy had 100% sensibility, 93% specificity, 88% positive predictive value and 100% negative predictive value [52], which adds encouragement to the PDOs platform as a decision-making tool. Furthermore, the authors compared response to the chemotherapy paclitaxel in sequential PDOs established prior to and after treatment in a patient diagnosed as initially paclitaxel-sensitive. In accordance with the clinical findings, PDOs derived from the responsive metastasis showed better sensitivity to paclitaxel compared with PDOs derived at progression from the same patient. Finally, the authors showed the potential to established in orthotopic human tumor xenograph models by implantation luciferase-expressing (Luc +) PDOs in the mouse liver (PDO-xenograph) followed by drug addition and evaluation. In this model, the effect of the regorafenib, a small molecule multikinase inhibitor against various pro-angiogenic and anti-proliferative targets [106], was tested for response from a patient with primary resistance and from a patient who achieved a durable response. In line with the clinical response, the PDOs xenographs from the regorafenib-sensitive patient displayed significant reduction in microvasculature presence in comparison with a non-significant change in the regorafenib-resistant patients. According with metastatic CRC-PDOs, Ooft and colleagues developed a method of testing treatment regimens in PDOs derived from metastatic liver lesions [38]. In this “TUMOROIDS” multicenter clinical study (NL49002.031.14) the authors tested in 10 patients and their respective CRC-PDOs the response to irinotecan monotherapy. The observed PDO drug response correlated with clinical outcome in 80% of patients by predictive performance using leave-one-out cross-validation (LOOCV). This correlation was specific to irinotecan-based monotherapy as, when the same methods were performed using combinate therapy of 5-FU plus oxaliplatin, the PDOs failed to predict treatment outcomes [38]. Complementing these findings, two different laboratories using rectal cancer PDOs presented preliminary studies which suggested prediction of response to chemoradiation [48, 63]. In this latter study, Ganesh and colleagues established organoids from a diverse patients set of rectal cancers (primary, metastatic and recurrent disease) and 21 different RC-PDOs were separately treated with 5-FU and FOLFOX. The data showed that RC-PDOs displayed heterogeneous outcomes in their response to clinical 5-FU and FOLFOX doses and in seven patients the in vitro response to combined 5-FU and FOLFOX treatment correlated with progression-free survival [63].

In a recent and highly promising study, Yao and colleagues, further reported 80 PDOs isolated from locally advanced rectal cancer treatment naïve patients enrolled in a phase III clinical trial that were subsequently treated with neoadjuvant chemoradiation (irradiation, 5-FU or CPT-11/irinotecan) [48]. The RC-PDOs were evaluated for individual treatments and when sensitivity was present in treated PDOs there was also clinical response in 68 of 80 patients (85%). The authors reported that these finding represented 84.43% correlation, 78.01% sensitivity, and 91.97% specificity [48].

In summary, the large-scale drug sensitivity screening in GI-PDOs can be performed within a window of 2–8 weeks [38, 44, 48, 52], whence this platform has the potential be used to guide or/and accelerate new clinical trials, and to suggest treatment recommendations within a clinically meaningful time frame. The contradictory and often lack of correlation between PDO response and clinical outcome may be the result of a number of variables. Firstly, the PDO reflects only a sub-section of the intratumoral heterogeneity that existed at the initiation of therapy [44]. Secondly, these models are limited by the absence of a complete microenvironment (eg. the patient immune system, microbiome and fibroblast components) and finally, the lack in vitro models of a liver does not allow for the variable of drug metabolism and thus the resulting changes in the concentration and time of exposure of circulating therapies that occur in the patient.

## Conclusions and future directions

PDOs hold the promise to be a significant player in the advancement of tumor biology. Suited to drug development, PDOs can be developed from healthy and diseased tissues to test for efficacy and toxicity, and most importantly, they have been shown to remain stable when expanded ex vivo. The FDA decision to modify the requirement that drugs in development must undergo animal testing before being administered to humans and receive approval, has opened the door to invest in the PDO culture [1, 2]. Potentially anticipating this decision, pharmaceutical companies decided in 2020 to phase out animal testing and, although they have not set a deadline for completion, all new drugs to be developed must feature alternatives to animal testing [107]. Currently, while there are several thousand patent applications that include the construction or use of organoids (Patent Public Database, www.ppubs.uspto.gov), there 107 clinical trials on the NIH clinical trials website (www.clinicaltrials.gov) using patient-derived organoids in evaluating therapy response. Only seven of these trials are currently closed, while the majority are in the stage of recruiting or registered to start recruiting. These clinical trials are almost exclusively in North America, Western/Northern Europe and China, and cover the majority of cancer types. This amount of clinical trials highlights the interest and the faith that the scientific community is placing in the use of organoids as a decision-making tool, and thus we await the results with high patient number and statistical power to enlighten us on the future path of this technology.

As an emerging research model, PDOs have achieved progress as a potentially predictive tool, due to their ability to recapitulate the phenotype and genetics of the organs of origin. To meet the criteria of offering "personalized” or “precision” therapy to patients these models need to be improved to incorporate the multifaceted aspects of the TME. Major questions remain unanswered and need further research. For example, how universal is the observation that primary cultures of PDO-forming cancer cells can be frozen and returned to culture with the formation of reproducible 3D structures and drug response? As reviewed herein, the current advances are heading in the correct direction and already offer the potential for PDOs complete with stromal, vasculature, microbiome and immune cell components. Also, these advances are improving the gap between 3D culture models and native ECM cues in tumor tissue, which is a crucial issue, considering that ECM is the major non-cellular stromal component in the TME and has a significant impact on cellular behavior. Ultimately the goal is to recreate the entire TME ex vivo to reduce animal use and lower drug screening costs. Most importantly is the still anecdotal potential that PDOs can offer clinical prediction to therapy choice**.** Already preliminary evidence is suggesting that once PDOs are established, the high-throughput drug screening in combination with DNA sequencing and/or other molecular or proteomic analysis techniques may help tailor cancer treatment to offer enhanced efficacy with lower side-effects and improved quality of life. To confirm any potential benefit in using PDOs for clinical prediction it is necessary to increase the PDOs sample size. While certain results to date may be promising, the conclusions drawn from low numbers of patients may be misleading. The scientific world experienced similar claims in the 1990s when MTS, MTT and ATP assays suggested that patient response to chemotherapy could be predicted from primary tumor cultured cells on plastic (culture-coated) plates, however these observations never withstood (in truth, never experienced) large scale preclinical trials. PDOs as a future clinical tool may not necessarily be restricted merely to drug administration and cancer cell viability; the field needs to be open to the incorporation of drug testing together with mutational signatures and markers of variability in components of the TME (eg. microbiome/immune cell presence) in potentially forming an algorithm to predict patient response to treatment ex vivo. Only time, and a lot of future testing, will reveal if the *holy grail of tissue culture* can be achieved and an ex vivo or in vivo model can give a reliably accurate prediction of patient drug response.

## Data Availability

Not applicable.

## Abbreviations

FDA: US Food and Drug Administration
PDO: Patient-derived organoid
GI: Gastrointestinal
CRC: Colorectal cancer
GC: Gastric cancer
HCC: Hepatocellular carcinoma
EAC: Esophagus cancer
PDAC: Pancreatic ductal adenocarcinoma
GBC: Gallbladder cancer
PDX: Patient-derived xerograph
2D: Two-dimensional
TME: Tumor microenvironment
3D: Three-dimensional
ECM: Extracellular matrix
CSC: Cancer stem cells
CAF: Cancer-associated fibroblasts
EMT: Epithelial-mesenchymal transition
WRS: Whole-exome sequencing
5-FU: 5-Fluorouracil
FOLFOX: 5-FU, leucovorin, oxaliplatin
FOLFIRI: Folinic acid, fluorouracil and irinotecan
NAT: Neoadjuvant chemotherapy
TCGA: The Cancer Genome Atlas Program
BTC: Biliary tract cancer
CCA: Cholangiocarcinoma
IHCC: Intrahepatic cholangiocarcinoma
CTLA-4: T-lymphocyte-associated protein 4
PD1: Programed death 1
PD-L: Programed death-ligand 1
MSI-H: Microsatellite instability-high
dMMR: Mismatch-repair-deficient dMMR
PBMC: Peripheral blood mononuclear cells
BME: Base matrix extracellular
RECIST: Response evaluation criteria in solid tumors
MHC: Major histocompatibility complex
AUC: Area under curve
LOOCV: Leave-one-out cross-validation
CPT-11: Irinotecan
